# Intergenerational Transmission of Trauma: The Mediating Effects of Family Health

**DOI:** 10.3390/ijerph19105944

**Published:** 2022-05-13

**Authors:** Emma M. Reese, Melissa Jane Barlow, Maddison Dillon, Sariah Villalon, Michael D. Barnes, AliceAnn Crandall

**Affiliations:** Department of Public Health, Brigham Young University, Provo, UT 84602, USA; melissajane.jbarlow@gmail.com (M.J.B.); dillonmadison@gmail.com (M.D.); sariahev@gmail.com (S.V.); michael_barnes@byu.edu (M.D.B.)

**Keywords:** adverse childhood experiences, adverse family experiences, positive childhood experiences, family health, intergenerational transmission of trauma

## Abstract

Family health is important to the well-being of individual family members and the collective family unit, and as such, may serve as a mediator for the intergenerational transmission of trauma (ITT). This study aimed to understand the intergenerational impact of parent’s adverse and positive childhood experiences (ACEs and PCEs) on their children’s adverse family experiences (AFEs) and how family health mediated those relationships. The sample consisted of 482 heterosexual married or cohabiting couples (dyads) in the United States who had a child between the ages of 3 and 13 years old. Each member of the dyad completed a survey, and data were analyzed using structural equation modeling. Parental ACEs were associated with more AFEs. The fathers’, but not the mothers’, ACEs were associated with worse family health. Parental PCEs were associated with better family health, and family health was associated with lower AFE scores. Indirect effects indicated that parental PCEs decreased AFEs through their impact on family health. Family health also mediated the relationship between the father’s ACEs and the child’s AFEs. Interventions designed to support family health may help decrease child AFEs.

## 1. Introduction

The American Psychological Association defines trauma as “an emotional response to a terrible event” [1]. Types of trauma are generally separated into two categories: interpersonal (e.g., abuse) and non-interpersonal (e.g., natural disasters or accidents) [2]. Childhood trauma subtypes vary in the research; however, childhood trauma scales typically include subsections for interpersonal trauma such as physical abuse, sexual abuse, emotional maltreatment, and neglect, and subsections for non-interpersonal trauma such as illness or death [3,4]. According to the Diagnostic and Statistical Manual of Mental Disorders, Fifth edition (DSM-V), individuals can experience trauma in four ways: by directly experiencing a traumatic event, witnessing a traumatic event, learning of a violent or accidental traumatic event that happened to a close family member or friend, or from extreme or repeated exposure to harsh details of a traumatic event [5].

Additionally, certain demographics, such as race, gender, and age, are important in the study of trauma. Individuals from non-White racial/ethnic backgrounds are more likely to experience trauma than White individuals are, demonstrating the importance of accounting for race in trauma research [6]. Results for gendered differences in experiencing trauma vary in existing research; however, some research identified gendered differences in number of trauma exposures and different types of trauma (e.g., exposure to domestic violence is more prevalent among males and sexual abuse is more prevalent among females) [6,7,8]. Additionally, research has identified gendered differences in trauma symptoms, specifically symptoms of post-traumatic stress disorder (PTSD) [7]. The age of first trauma is particularly important, as it can impact healthy development; for example, individuals who are extremely traumatized typically experience trauma earlier in childhood than those with less trauma [9].

Trauma experienced in childhood has significant implications for healthy child development and psychopathology in adulthood [3,4,6,9]. Brain imaging studies have shown that all types of childhood trauma are related to decreased volume of the frontal cortex, an area of the brain associated with reasoning, emotion, and language [4]. Essentially, the traumatic stressors children experience cause brain injury, increasing the risk of psychopathology in adulthood [4]. Epidemiological studies often focus on common mental disorders as outcomes of childhood trauma, including internalizing psychopathology (e.g., mood and anxiety disorders) and externalizing psychopathology (e.g., substance use disorders) [6]. A parent’s psychopathology can increase the risk of childhood trauma among their children, thus revealing the cyclical nature of trauma transmission between parents and children [6].

Families play a central role in child development and in the intergenerational transmission of trauma (ITT) [6]. Factors that impact ITT include family functioning, parenting ability, parent–child relationship quality, cognitive appraisal of trauma, PTSD, and severity of a parent’s childhood trauma [10,11,12,13,14]. Trauma (including childhood trauma) experienced by parents can negatively affect their parenting ability while also increasing the risk of transmitting trauma to their children [14]. The purpose of this study is to examine how parents’ trauma, experienced in childhood, measured through adverse childhood experiences (ACEs), when also accounting for their positive childhood experiences (PCEs), affects their children’s experiences of trauma, as measured through adverse family experiences (AFEs). A second purpose is to examine whether the family’s health mediates the relationship between childhood experiences and later childhood trauma.

### 1.1. Risk Factors for Adverse Family Experiences

Measures of childhood trauma, such as ACEs and AFEs, are key to understanding ITT. ACEs measure an individual’s cumulative experience of various forms of abuse, neglect, and household dysfunction during their childhood, which can be potentially traumatic [15,16,17]. ACEs are related to behavioral and health problems in adulthood [15,18]. Studies about the role of ACEs in the intergenerational and community transmission of trauma have primarily focused on large-scale trauma such as the Holocaust; PTSD among veterans of war; and historical, systematic, and generational trauma [19,20,21,22]. However, as trauma research has developed, ACEs and trauma are now being applied to the general population. Previous findings indicate that parents who experience trauma have diminished capabilities to empathize with their child’s emotions due to an altered perception of the world and the individuals they interact with [19,20]. Diminished parenting skills may result in decreased trust and feelings of safety for their children from a lack of emotional stability [20]. In turn, children mirror their parents’ instability, and the process of ITT continues.

AFEs items were derived from ACE questionnaires but use an environmental perspective regarding family or household dysfunction and risk factors in the family unit [23]; AFEs introduced four new items in addition to five derived ACE items [24,25]. While AFE items are similar to ACE items, there are two important differences: AFEs do not include questions about the child’s personal experience of physical, emotional, or sexual abuse by guardians or caregivers, and parents respond about their child’s experience rather than their own [25]. AFEs provide more understanding of the child’s environmental experience; however, parental ACEs may determine how AFEs exist in families. Exposure to AFEs is associated with negative childhood development, including decreased physical health, mental health, and well-being [26]. A poor-quality, high conflict, and unsupportive family environment may cause chronic stress to the entire family [27]. Empirical evidence confirms that the family environment is critical to shaping childhood development, perhaps more than any other environment.

### 1.2. Promotive Factors for Adverse Family Experiences

Recent research has examined the role of cumulative positive or promotive experiences in childhood, including benevolent childhood experiences (BCEs) [28], positive childhood experiences (PCEs) [29], advantageous childhood experiences (counter-ACEs) [30], and various resilience questionnaires and frameworks. Positive experiences during childhood have been shown to promote better adult health even in the presence of high ACEs [28,29,30].

In addition to PCEs, a healthy family environment in adulthood may be important to reducing children’s AFEs. Family health is defined as “a resource at the level of the family unit that develops from the intersection of the health of each family member, their interactions and capacities, as well as the family’s physical, social, emotional, economic, and medical resources” [31]. Better family health could also be viewed as an advantageous family experience, or “counter-AFE”, as it helps to increase the positive experiences of a whole family. However, until the recent creation of the Family Health Scale, there were few measures of family health [32]. In research using the Family Health Scale, parental PCEs promote better family health, whereas their history of ACEs may harm the family’s health [33]. Other studies have shown that healthy family functioning (which is related to family health, but focused on family routines and habits) can help increase the positive development of children in the family. This includes joint family activities, healthy parental supervision, and healthy interactions in the family [26]. Thus, family health may be a pathway through which parental childhood experiences influence whether trauma is transmitted to the child.

### 1.3. Family Systems Theory and ITT

The family unit is a unique organizing structure of health and well-being, and during a crisis, it may serve as an important mediator for children’s risk and resilience [34]. Family systems theory provides a framework to understand the role of family health in ITT. Family systems theory posits that family members are interdependent, and one family member’s well-being can have a significant impact on another member’s well-being [13]. Understanding this interdependence can help explain the cyclical nature of ITT—specifically how childhood trauma can determine later relationship quality with partners and children, which can then increase family dysfunction via negative relationship quality [13]. Thus, family systems theory represents the interdependence of trauma transmission between all family members.

### 1.4. Aims and Purpose

There is a gap in the literature regarding family health as a mediator between childhood experiences and AFEs and understanding the relationship between parental PCEs and child AFEs. Therefore, the purpose of this study is to understand the intergenerational impact of parent ACEs and PCEs on their children’s AFE scores, and how family health mediates those relationships. Specifically, this study aims to answer the following research questions: (1) Do parent’s adverse and positive childhood experiences predict their children’s AFEs? We hypothesized that there would be a positive relationship between both the mother’s and father’s ACEs and the child’s AFEs, and an inverse relationship between the mother’s and father’s PCEs and the child’s AFEs. (2) Does family health mediate the relationship between parent’s childhood experiences and children’s AFEs? We hypothesized that family health would mediate the relationship between childhood experiences and AFEs. This study is important because it considers family health as a mediator in the intergenerational transmission of trauma by considering the roles of both adverse and positive childhood experiences in the family health of the second-generation household.

## 2. Materials and Methods

### 2.1. Sampling and Procedures

The sample consisted of 482 married or cohabitating couples (dyads) who were living in the United States at the time of the survey. Each couple had a child between the ages of 3 to 13 years old. Only heterosexual dyads were included in this study due to a low response rate from same-sex dyads. The sample was recruited via a Qualtrics panel. To obtain a more representative sample, a proportion of the sample was required to have at least one partner in the dyad who was a racial minority or at least one partner who had less than a high school degree. Each member of the dyad completed a 20 min survey. Approval for this study came from the Brigham Young University Institutional Review Board (IRB), and all participants were compensated with Qualtrics credits. Compensation varied for participants based on the difficulty to recruit certain participants, especially those from minority groups.

### 2.2. Measures

#### 2.2.1. Adverse Family Experiences

AFEs were measured using the 9-item AFE module from the National Survey of Children’s Health 2011–12 [23]. The AFE items were a subset of the Behavioral Risk Factor Surveillance System (BRFSS) ACE Module created to measure a family-oriented perspective of adverse experiences during childhood. Mothers responded to the questions about their oldest child who was between 3 and 13 years. The items examined family dysfunction and risk factors within the family unit, such as “Did your child ever live with anyone who was mentally ill or suicidal, or severely depressed for more than a couple of weeks?” and “Was your child ever the victim of violence or witness any violence in [his/her] neighborhood?” and “Since your child was born, how often has it been very hard to get by on your family’s income, for example, it was hard to cover the basics like food and/or housing”. [23]. Response options for seven of the nine items were in a dichotomous *Yes* (coded as 1) *No* (coded as 0) format, while the other items were on a 4-point frequency scale from *Never* to *Very often*. *Never* and *Not very often* responses were coded as 0, and *Somewhat often* and *Very often* responses were coded as 1. Responses were summed to create a cumulative score ranging from 0 to 9.

#### 2.2.2. Adverse Childhood Experiences Questionnaire

The ACE items were derived from the BRFSS ACE module [17]. BRFSS annually gathers data through states in the U.S. by random telephone surveys [17]. The ACE module includes 11 items that measure risk factors prior to the age of 18. Constructs of the items include various forms of abuse, neglect, and general household dysfunction, such as “Did you live with anyone who was depressed, mentally ill, or suicidal?” and “Were your parents separated or divorced?” and “How often did anyone at least 5 years older than you or an adult, force you to have sex?” [15,16,17]. Response options include *Yes*, *No*, and *I don’t know* for each item. The *Yes* responses were summed to create a cumulative score ranging from 0 to 8. Separate scores were created for mothers’ and fathers’ ACEs.

#### 2.2.3. Family Health Scale—Short Form

The FHS-SF is a 10-item scale created to measure overall family health, with sample items including: “We support each other”, “We help each other make healthy changes”, and “My family did not have enough money at the end of the month after bills were paid”. [32]. Response options were recorded on a 5-point Likert scale ranging from *Strongly agree* to *Strongly disagree*, and negatively worded items were reverse coded. Responses were summed to create a cumulative score ranging from 0 to 10. Both parents reported on their family’s health in adulthood at the time of the study. The FHS-SF measure was included in the final models as a single latent variable comprising both partners’ responses. Prior research indicated that the scale is most reliable as a single measure with multiple reporters from the same family rather than using separate measures for each responder and has a Cronbach’s alpha of 0.88 when including responses from both partners [35].

#### 2.2.4. Positive Childhood Experiences

PCEs were measured using the 10-item BCE questionnaire [28], and 3 items from the PCE questionnaire [29]. Key themes of these items include social support, perceived safety, and positive and stable quality of life. Sample items included: “Did you have at least one caregiver with whom you felt safe?” and “Did you have beliefs that gave you comfort?” and “Were you able to talk with your family about your feelings?” [28,29]. Response options included *Yes* or *No*. The *Yes* responses were summed to create a cumulative score ranging from 0 to 13, with a higher score equaling more PCEs. Separate scores were created for mothers’ and fathers’ PCEs.

### 2.3. Data Analysis

Data were cleaned, and item distributions were analyzed using Stata 17. A structural equation modeling framework was used to examine model relationships. Family health was included in the measurement model as a latent variable and showed good model fit based on the root mean square error of approximation (RMSEA = 0.051) and comparative fit index (CFI = 0.981). A structural model was fit by regressing child AFEs on family health, father’s ACE score, mother’s ACE score, father’s PCE score, and mother’s PCE score. Family health was regressed on the mother’s and father’s ACEs and PCEs. Controls (child’s age, child’s gender, mother’s age, and mother’s race) were added to the final model by regressing all covariates of interest on the demographic controls. Model fit was examined using the following model fit cutoffs: RMSEA < 0.08 and CFI > 0.90 indicated adequate fit [36,37,38]. Indirect effects were examined using 5000 bootstraps to ensure robust standard errors [39]. All models were estimated using robust weighted least squares, which is appropriate for categorical data. Missing data were minimal (<1% across all items), and full information maximum likelihood (FIML) was used to account for missing items. The results presented include standardized betas.

## 3. Results

Most participants (90.4%) were married, and 12% were in an interracial relationship. The mothers’ mean age was 35.6 years. About 73.4% of the mothers were White, and 14.11% had a high school education or less. The fathers’ mean age was 38.9 years. About 74.9% of the fathers were White, and 17.01% of fathers had a high school education or less. Mean ACE scores for mothers and fathers were 2.1 and 2.08, respectively, while mean PCE scores were 10.98 and 10.91, respectively. The children’s gender distribution was 41.7% female and 58.3% male, and the average age of children reported was 9.75 years old. Lastly, the average child AFE score was 0.92 (see Table 1 for full descriptive results of the sample).

**Figure 1:**
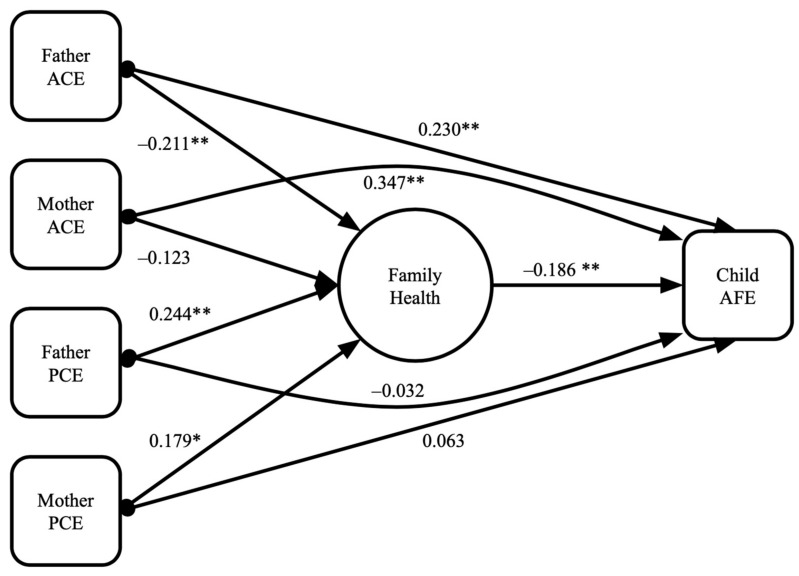
Structural equation model: Parental ACE and PCE associations with family health and child AFE. Notes: RMSEA = 0.042; CFI = 0.963. * *p* < 0.05, ** *p* < 0.001.

The final model (Figure 1) had good model fit (RMSEA = 0.042; CFI = 0.963). Both fathers’ ACEs and mothers’ ACEs were associated with an increased number of child AFEs. Fathers’, but not mothers’, ACEs were associated with worse family health. Mothers’ and fathers’ PCEs were not directly associated with AFEs. Both mothers’ and fathers’ PCEs were associated with better family health. Family health was associated with lower AFE scores. Indirect effects indicated that parental PCEs decreased AFEs by impacting family health. Family health also mediated the relationship between fathers’ ACEs and children’s AFEs. However, family health did not significantly mediate the relationship between mothers’ ACEs and children’s AFEs (Table 2).

## 4. Discussion

The results confirmed intergenerational transmission of trauma (ITT) from both mothers and fathers to their children, especially via the relationship between mothers’ ACEs and children’s AFEs (consistent with hypothesis one). Contrary to our first hypothesis, parental PCEs were not directly related to the child’s AFEs. Fathers’ ACEs were predictive of worse family health, but there was no association between mothers’ ACEs and family health. Both mothers’ and fathers’ PCEs were predictive of positive family health. Further, consistent with hypothesis two, family health mediated the relationships between both parents’ PCEs and father’s ACEs with the child’s AFEs. The findings support the intergenerational transmission of ACEs from both mothers and fathers to their children and are consistent with previous findings on the intergenerational transmission of ACEs [14], but they also support the mediating role of positive effects in childhood and adulthood in reducing ITT.

### 4.1. Fathers’ ACEs and Family Health

Fathers’ ACEs were predictive of worse family health more so than mothers’ ACEs. Fewer studies exist about the effects of fathers’ ACEs in family functioning. Existing research demonstrates that genetics and environment have a strong influence on ACE exposure among males, while female exposure was largely driven by environment [40]. Given that ACEs increase risk of psychopathology in adults [6], research also shows that men are more likely to experience externalizing symptoms of psychopathology such as substance abuse or reckless activities [40,41]. Both mental illness and externalizing symptoms may harm the family’s social and emotional health processes and resources, undermining their family health. Research further indicates that the parenting styles of fathers are related to the perceived harsh parenting style that the father was raised under in his childhood [42]. This result may occur because psychological distress mediates the relationship between higher ACE scores and fathering behavior [43]. Additionally, the socialization of gendered parenting roles may influence the impact of either parent’s ACEs on their parenting ability; for example, fathers’ ACEs decrease instrumental (e.g., caregiving) and expressive (e.g., emotional support) parenting ability, while research on mother’s ACEs influence on instrumental parenting typically has found no relationship [43].

Generally, maternal mental health is screened and monitored regularly during prenatal and postnatal healthcare. Pediatricians often focus on the mother–child dyad as the key determinant of family health [41]; however, creating a mother–father–child triad may facilitate a more family-centered approach to AFE prevention. Therefore, a potential intervention is to include fathers in pediatric visits and postnatal mental health assessments. Pediatricians may increase fathers’ involvement by addressing both the mother and father during appointments, assessing relationship health between parents, demonstrating the value of fathers at clinical practices, and educating fathers on childcare and parenting [41]. Given that fathers’ ACEs were found to be more indicative of family health status, the integration and development of fathers’ mental health resources could be a tool to improve family health [41,44].

### 4.2. Parental PCEs and Family Health

While mother’s and father’s PCEs did not have significant direct relationships with the child’s AFEs, both parents’ PCEs were associated with better family health, which was negatively related to the child’s AFEs. This indicates that PCEs may decrease ITT from parents to children through family health. The fathers’ PCEs were more strongly associated with higher levels of family health compared to the mothers’ PCEs, which is supported by evidence showing that increased involvement from fathers mitigates poor physical outcomes in children, such as obesity rates, cognitive development, and ITT [45,46]. Strong family health can include access to physical, social, emotional, financial, and medical resources; healthy habits; strong emotional and social health processes; and external social support [32]. According to the findings of the current study, PCEs were predictive of these resources within families. Additionally, the framework of family systems theory supports existing evidence of the relationships between PCEs and family health. Previous research demonstrated that PCEs more significantly influenced family health than ACEs [33]. The quality of marital relationships in families may also reduce the parent–child transmission of trauma, especially from parents with posttraumatic and secondary traumatic stress symptoms [11]. Therefore, family environments that are low in conflict and have high emotional support are promotive factors that help reduce the child’s AFEs [27]. With the apparent interconnectedness of the family unit, promotive factors such as PCEs and good family health may help to reduce AFEs.

### 4.3. Family Health as a Mediating Factor

Lastly, consistent with family systems theory, family health mediated the relationship between the father’s ACEs and their child’s AFEs as well as between both parents’ PCEs and the child’s AFEs. Based on the results of this study, improving family health may help reduce ITT. For example, the COVID-19 pandemic provided a unique opportunity to study disruptions to the family system such as stressors and traumatic experiences caused by infectious disease and government restrictions. In a summary of articles about family dynamics during COVID-19, findings suggest that the pandemic disproportionately affected at-risk individuals and families, specifically among those with lower-quality family relationships and limited resources [47]. Apart from COVID-19 stressors and trauma, lower-SES children are at greater risk of experiencing environmental trauma from lack of resources, which then increases their risk of psychopathology [48]. Previous research has identified parent psychopathology as a risk factor for their children experiencing trauma [6]. Since low SES and experiencing trauma (such as AFEs) are risk factors for psychopathology (a risk for childhood trauma), interventions targeting lower SES or disadvantaged families may help improve family health, especially since AFEs are more common among lower-income families [49].

Involving the entire family to strengthen family systems could improve family health and functioning as well as reduce ITT [50]. Family health interventions exist at the primary, secondary, and tertiary prevention level, and given the heritable nature of trauma, secondary or tertiary interventions for parents may serve as primary prevention for trauma transmission to their children. Successful primary prevention examples include home visits from social service professionals and community health workers [51,52], and community-based coalitions such as the Communities That Care system, which uses community needs assessments to address risk and protective factors within a community that emphasize child resilience such as school and sport programs and other community opportunities that support healthy parent and child interactions through local programs such as parent training, community gardening, or community cooking classes [51]. A secondary prevention such as the 2021 American Families Plan from the Biden administration impacts low-SES individuals and families. This plan focuses on improving the economic well-being of U.S. residents by increasing the child tax credit and access to affordable education [53]. Improving the economic well-being may decrease the risk of children experiencing trauma, thus reducing ITT. Another example, which can act as a primary, secondary, or tertiary prevention method is the Family Empowerment Program—a therapy program that focuses on the family system by partnering with an interdisciplinary team of professionals who provide the family with resources and treatment [54]. Additionally, family-friendly workplace policies allow employees to fulfill their family and work obligations through telecommuting, flexible time off, and paid childcare. Future studies should examine promising family-friendly practices shown to increase motivation and productivity in the workforce for both men and women and should incorporate AFE and other risk measures across a wide spectrum of worksites, including many small business and service industries which are less likely to offer such benefits. All levels of prevention targeting family health are key to reducing the risk of AFEs and transmission of trauma from parents to children.

### 4.4. Limitations

This study had several limitations, some of which were due to the circumstances of the COVID-19 pandemic. The situational effects of COVID-19 such as quarantine and social isolation may have affected the report of PCEs, ACEs, and AFEs reported in this study. An additional limitation was the low variability in PCE scores among the couple dyads, which prevented us from examining PCEs as a moderating variable. Another limitation based on the methodology of the study was that the AFE information was based on the mothers’ reports rather than on direct reports from the children. Parents may be less likely to report adversity in their children’s lives and may be a less accurate representation of a child’s perception of family life [55]. While future research may determine AFE reports directly from children, our study focused on young children who may have been too young to answer for themselves. In the current study, only mothers’ report of AFEs was used because in a few cases fathers and mothers reported on different children. Recent research indicates that mothers typically fill the responsive or nurturing role in heterosexual relationships and may be more aware of their children’s potentially traumatic experiences, while fathers more often take a protective role [24,56]. However, the mothers may have reported their child’s AFEs differently from the fathers’ reports, which may have affected the results. Future research accounting for both mothers’ and fathers’ responses to AFE items is important. Additionally, the ACE and AFE measures used do not represent all forms of trauma, such as homelessness. Finally, the data were cross-sectional limiting the ability to establish causality between family health and the children’s AFEs.

## 5. Conclusions and Future Research Directions

This research provides further implications for promoting advantageous childhood and family experiences through family health. Further research is needed to build on the results found in the current study. Since the results were likely affected by the ongoing COVID-19 pandemic, a replication study should be conducted post-pandemic to determine the generalizability of the results in less stressful conditions. A post-pandemic study might demonstrate the pathways between family health and ITT without the constant stress of a worldwide pandemic, while further contributing research on the impact of pandemic stressors and trauma on family health and transmission of trauma. Additionally, longitudinal data would be imperative to confirming directionality of results and the impact of adverse and positive childhood experiences on family health over time. Future research focusing on differences in mothers’ and fathers’ ACE transmission could provide a better understanding of why fathers’ experiences were more impactful and how father-focused interventions may promote better family health. This research could also identify differences in ACEs and family health in households where one or both parents experienced ACEs. The results of this study indicate that family health does mediate the relationship between the fathers’ ACEs and the children’s AFEs, demonstrating the necessity of interventions to promote family health and inhibit the intergenerational transmission of ACEs.

## Figures and Tables

**Table 1:** Sample Demographics.

Mean child age (years)	9.75	
Marital status %		
Married	90.4	
Cohabitating	9.6	
Interracial relationship	12	
	Female	Male
Child gender %	41.7	58.3
Mean parent age	35.6	38.9
Race (White) %	74.9	73.4
Education (high school or less) %	14.1	17.01
Average ACE scores (range 0–8)	2.1	2.08
Average PCE score (range 0–13)	10.98	10.91
Average child AFE scores (range 0–9)	0.92	

**Table 2:** Significant family-health-mediated indirect pathways.

Indirect Pathways	Beta	Z-Score	*p*-Value
Father’s ACE → Family Health → Child’s AFE	0.039	2.460	0.014
Mother’s ACE → Family Health → Child’s AFE	0.023	1.754	0.079
Father’s PCE → Family Health → Child’s AFE	−0.045	−2.904	0.004
Mother’s PCE → Family Health → Child’s AFE	−0.033	−2.470	0.014

## Data Availability

Data are not publicly available due to IRB protocols for the study.
